# The Role of NMDA Receptor Subtypes in Short-Term Plasticity in the Rat Entorhinal Cortex

**DOI:** 10.1155/2008/872456

**Published:** 2008-10-29

**Authors:** Sophie E. L. Chamberlain, Jian Yang, Roland S. G. Jones

**Affiliations:** Department of Pharmacy and Pharmacology, University of Bath, Claverton Down, Bath BA2 7AY, UK

## Abstract

We have previously shown that spontaneous release of glutamate in the entorhinal cortex (EC) is tonically facilitated via activation of presynaptic NMDA receptors (NMDAr) containing the NR2B subunit. Here we show that the same receptors mediate short-term plasticity manifested by frequency-dependent facilitation of evoked glutamate release at these synapses. Whole-cell patch-clamp recordings were made from layer V pyramidal neurones in rat EC slices. Evoked excitatory postsynaptic currents showed strong facilitation at relatively low frequencies (3 Hz) of activation. Facilitation was abolished by an NR2B-selective blocker (Ro 25-6981), but unaffected by NR2A-selective antagonists (Zn^2+^, NVP-AAM077). In contrast, postsynaptic NMDAr-mediated responses could be reduced by subunit-selective concentrations of all three antagonists. The data suggest that NMDAr involved in presynaptic plasticity in layer V are exclusively NR1/NR2B diheteromers, whilst postsynaptically they are probably a mixture of NR1/NR2A, NR1/NR2B diheteromers and NR1/NR2A/NR2B triheteromeric receptors.

## 1. INTRODUCTION

A huge amount of research has been devoted to the study of the physiology, pharmacology, function, and pathology of NMDA receptors (NMDAr). This has been extensively reviewed elsewhere (e.g., [1–6]). Native NMDAr are heteromeric structures, and consist of NR1 subunits, which are obligatory, in combination with one or more of four subtypes of NR2 subunit (NR2A-D). Functional receptors are tetramers, comprising two NR1 subunits and two NR2 subunits, where the functional unit is probably an NR1/NR2 heterodimer. The functional properties of NMDAr, such as single channel conductance, the degree of voltage-dependent Mg^2+^ block, and deactivation kinetics depend on which of the four NR2 subunits is assembled in the receptor. For example, NR2A and NR2B-containing channels have a high single channel conductance (40–50 pS) whereas NR2C and NR2D are lower (15–35 pS). NR2A-containing receptors display fast decay kinetics (around 100 milliseconds), whereas NR2B and C are much slower (250 milliseconds), and NR2D slower still (4 seconds) [5, 7]. In addition to functional differences, various subunit combinations display pharmacological differences in susceptibility to antagonists and regulatory mechanisms (such as sensitivity to H^+^, Zn^2+^, polyamines).

Synaptic transmission is a highly dynamic and plastic process, modified on-demand by a myriad of instantaneous, short, intermediate, and long-term regulatory mechanisms. Much attention has been devoted to the study of the role of NMDAr in synaptic plasticity, particularly in long-term potentiation (LTP) and depression (LTD). These studies have largely focussed on NMDAr at postsynaptic sites. However, dynamic regulation of synaptic strength can also involve receptors on presynaptic terminals, which provide a powerful, synapse-delimited control of transmitter release, and the existence of presynaptic NMDAr (preNMDAr) is now firmly established. Neurochemical [8–11] and immunolocalization studies [12–15] provided early indications for preNMDAr. We provided the first clear functional demonstration of preNMDAr, showing that the competitive antagonist, 2-AP5, could reduce the frequency of spontaneous excitatory postsynaptic currents (sEPSCs) at glutamate synapse in the rat entorhinal cortex (EC), indicating a tonic facilitatory effect of preNMDAr on glutamate release [16]. PreNMDAr are now known to modify both glutamate and GABA release in a wide variety of locations and tissues [17–33].

Increasing attention is being paid to the role of preNMDAr as mediators of both long-term alterations in synaptic strength, and in moment-to-moment and short-term activity-dependent changes in transmitter release. For example, a role of preNMDAr in LTD has been demonstrated in cerebellum [34], visual [22, 33], and somatosensory [17] cortex. Conversely, involvement of preNMDAr in LTP has been demonstrated in amygdala [26, 32]. More intermediate forms of potentiation of glutamate [30] and GABA transmission [23], over a time scale of minutes, may also involve preNMDAr. As noted above, we found that preNMDAr are tonically activated by ambient glutamate [17, 35], providing instantaneous control over the level of glutamate release at EC synapses. Similar results have been reported for other areas [22, 27, 28, 33]. In addition, we found that preNMDAr are activated after action potential-driven synaptic release of glutamate, increasing the probability of subsequent release and allowing them to mediate short-term, frequency-dependent facilitation of glutamate transmission [16, 35].

We have also demonstrated that the tonic facilitatory effect of preNMDAr on spontaneous glutamate release is likely to be predominantly mediated by NR2B-containing NMDAr, since the increase induced by 2-AP5 was mimicked [35, 36] by relatively specific blockers of the NR2B subunit, ifenprodil [37], and Ro 25-6981 [38]. In addition, an antagonist with some specificity (albeit weak) for the NR2A subunits, NVP-AAM077 [39] had little effect. Others have also concluded that preNMDAr are likely to be predominantly NR2B-containing [27, 33, 40]. Postsynaptically, both NR2A and NR2B contribute to glutamate transmission, although there is controversy over whether diheteromeric NR1/NR2A and NR1/NR2B coexist at the postsynaptic density, or are segregated between synaptic and extrasynaptic locations, or even in a synapse-specific way [3]. The contribution of triheteromeric NR1/NR2A/NR2B receptors is also still a matter of debate [3, 41].

In the present study, we have extended our studies in the EC to examine the contribution of NR2A and NR2B receptors to short-term plasticity of glutamate transmission, by examining the effects of relatively specific blockers on the preNMDAr mediated, frequency-dependent facilitation of evoked glutamate release. In addition, we have used the same agents to determine whether postsynaptic NMDAr may differ from those on presynaptic terminals.

## 2. METHODS

### 2.1. Slice preparation

Experiments were performed in accordance with the U.K. Animals (Scientific Procedures) Act 1986, European Communities Council Directive 1986 (86/609/EEC), and the University of Bath ethical review document. Slices containing EC and hippocampus were prepared from male Wistar rats (P28–35), which were anaesthetized with an intramuscular injection of ketamine (120 mg/kg) plus xylazine (8 mg/kg) and decapitated. The brain was rapidly removed and immersed in oxygenated artificial cerebrospinal fluid (aCSF) chilled to 4°C. Slices (350–400 *μ*m) were cut using a Vibroslice, and stored in aCSF bubbled with 95% O_2_/5% CO_2_, at room temperature. Following recovery for at least 1 hour, individual slices were transferred to a recording chamber mounted on the stage of a Zeiss Axioskop FS or an Olympus BX50WI microscope. The chamber was perfused (2.0 ml/min) with oxygenated aCSF (pH 7.4) at 31–33°C. The aCSF contained (in mM) NaCl (126), KCl (3), NaH_2_PO_4_ (1.4), NaHCO_3_ (19), MgSO_4_ (2), CaCl_2_ (2), and D-glucose (10). Neurones were visualized using differential interference contrast optics and an infrared video camera.

### 2.2. Electrophysiological recording

Patch pipettes were pulled from borosilicate glass on a Flaming/Brown microelectrode puller. For recording spontaneous (sEPSCs) or evoked (eEPSCs) excitatory postsynaptic currents, pipettes were filled with a Cs-gluconate-based solution containing (in mM) D-Gluconate (100), HEPES (40), QX-314 (1), EGTA (0.6), NaCl (2), MgCl_2_ (5), TEA-Cl (1), phosphocreatinine (5); ATP-Na (4), GTP-Na (0.3), MK-801 (2). Solutions were adjusted to 290 mOsmol, and to pH 7.3 with CsOH. Whole-cell voltage clamp recordings (holding potential −60 mV unless otherwise stated) were made from neurones in layer V of the medial division of the EC, using an Axopatch 200B amplifier (Molecular Devices, Calif., USA). Series resistance compensation was not employed, but access resistance (10–30 MΩ) was monitored at regular intervals throughout each recording and cells were discarded from analysis if it changed by more than ±10%. Liquid junction potential (12.3 mV) was estimated using the Junction Potential Calculator included in pClamp-8 software (Molecular Devices, Calif., USA), and compensated for in the holding potentials.

eEPSCs were elicited by electrical stimulation (bipolar pulses, 10–50 V, 0.02 millisecond duration) via a bipolar tungsten electrode placed on the surface of the slice in layer V of the lateral EC. The stimulation intensity was adjusted to give submaximal (approx. 50–60% maximum amplitude) responses.

### 2.3. Monitoring presynaptic NMDAr activity

In all these experiments, MK-801 (2 mM) was included in the patch pipette solution to block postsynaptic NMDAr. This allowed us to record AMPA-receptor mediated responses in isolation, and to monitor activity at preNMDAr uncontaminated by postsynaptic receptor effects. This approach was developed by us [16, 35, 42], and has been used successfully by others to block postsynaptic NMDAr in the recorded neurone [17, 27, 28, 32, 33, 40]. When whole-cell access was gained, neurones were voltage clamped at 0 mV, and synaptic stimulation was delivered at 2 Hz for 30–40 seconds to allow blockade of postsynaptic NMDAr by MK-801 dialyzed into the cell via the patch pipette solution. Membrane potential was then clamped at −60 mV and single shock stimulation delivered at low frequency (0.05 Hz) to evoke AMPAr mediated EPSCs. At 2 or 3 minute intervals, the single shock was replaced with stimulation at 3 Hz for 10 seconds. Such stimulation results in a frequency-dependent facilitation of the AMPAr-mediated EPSC, which we have shown previously to be dependent on activation of preNMDAr [35]. We used the degree of frequency-dependent facilitation of AMPAr-mediated eEPSCs as a quantitative measure of preNMDAr activation.

### 2.4. Monitoring postsynaptic NMDAr activity

In these experiments, MK-801 was omitted from the patch pipette solution. When whole-cell access was gained, control eEPSCs were recorded at a holding potential of −60 mV, before addition of the AMPAr antagonist, NBQX, and the GABA_A_r-antagonist, bicuculline to the bath perfusion. After 10–12 minutes, the holding potential was changed to +40 mV to record isolated NMDAr-mediated EPSCs as positive going currents. These were evoked at low frequency (0.05 Hz) until stable amplitudes were recorded, before addition of antagonists to the bath.

### 2.5. Data analysis

Data were recorded to computer hard disk using Axoscope software. Minianalysis (Synaptosoft, Decatur, Ga, USA) was used for analysis of EPSCs offline. In the studies of preNMDAr, the average peak amplitude of the 8 responses before each episode of 3 Hz stimulation was determined. During the period of 3 Hz stimulation, the amplitude of the 8 largest events was determined and normalized to the average amplitude of the preceding low-frequency events to obtain a quantitative measure of frequency-dependent facilitation in the presence and absence of antagonists. In these studies, we also analyzed AMPAr-mediated sEPSCs, by determining interevent interval (IEI), amplitude, rise (10–90%), and decay times. sEPSCs were detected automatically using a threshold-crossing algorithm. Threshold varied from neurone to neurone but was always maintained at a constant level in any given recording. At least 200 events were sampled during a continuous recording period for each neurone under each condition. Cumulative probability distributions of IEI were compared using the Kolmogorov-Smirnoff test. In experiments on postsynaptic NMDAr, responses were quantified by measuring mean peak amplitudes of at least 5 NMDAr-mediated eEPSCs evoked at low frequency at intervals throughout the study. In these studies, the vast majority of sEPSCs were blocked, as recordings were conducted in the presence of NBQX. Occasional slow sEPSCs mediated by NMDAr were recorded, their frequency was very low (2-3 per minute) and precluded meaningful analysis.

### 2.6. Materials

Salts used in preparation of aCSF were “Analar” grade and purchased from Merck/BDH or Fisher Scientific (Dorset, UK). All drugs were applied by bath perfusion. MK-801, NMDA, NBQX, D-2-AP5, bicuculline methiodide, and Ro 25-6981 ((*α*R,*β*S)-*α*-(4-hydroxyphenyl)-*β*-methyl-4-(phenylmethyl)-1-piperidinepropanol hydrochloride) were obtained from Tocris (Bristol, UK). TPEN (N,N,N′, N′-Tetrakis-(2-pyridylmethyl)-Ethylenediamine) was obtained from Sigma (UK). UBP302 ((S)-1-(2-amino-2-carboxyethyl)-3-(2-carboxybenzyl) pyrimidine-2,4-dione) was a kind gift from Dr. Dave Jane, University of Bristol, and NVP-AAM077 ((R)-[(S)-1-(4-bromo-phenyl)-ethylamino]-(2,3-dioxo-1,2,3,4-tetrahydroquinoxalin-5-yl)-methyl]-phosphonic acid) was a gift from Dr. Yve Auberson at Novartis (Basel, Switzerland).

## 3. RESULTS

### 3.1. Presynaptic NMDAar

**Figure 1:**
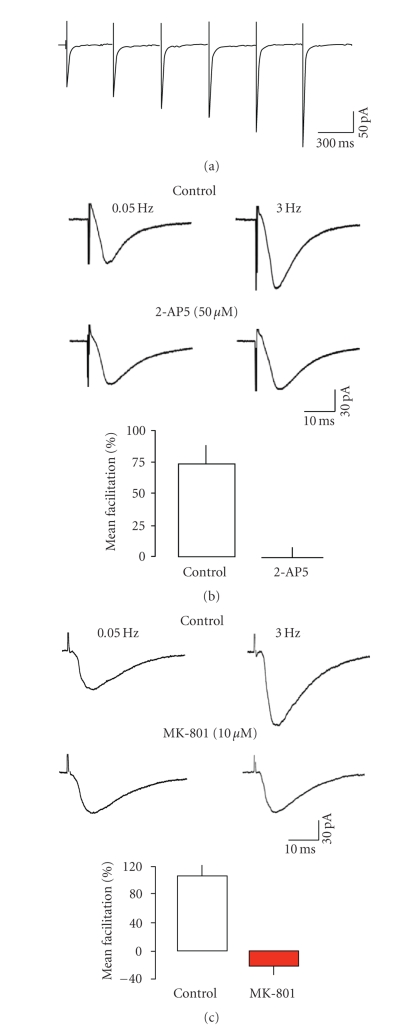
Short-term facilitation is mediated by presynaptic NMDA receptors. (a) First 6 responses evoked by a train of stimuli (3 Hz, 20 seconds) averaged from 3 neurones. (b) Responses (*n* = 8) were averaged at low frequency and during 3 Hz stimulation. In the presence of 2-AP5, low-frequency responses were unaltered, but facilitation was abolished. The bar graphs show the mean results from 5 neurones. (c) Similar results were seen with MK-801. Stimulation artifacts have been partially blanked for clarity.

Figure 1(a) shows eEPSCs evoked in a layer V neurone at 3 Hz, with postsynaptic NMDAr blocked by internally dialyzed MK-801. The first 6 responses evoked during a train of 30 at 3 Hz are shown and demonstrate the facilitation seen at this relatively low frequency. As reported previously [35], the facilitation of the AMPAr-mediated eEPSCs was entirely dependent on presynaptic NMDAar activation, since it could be abolished by 2-AP5 (*n* = 5, Figure 1(b)). Likewise, the NMDAr channel blocker, MK-801, also abolished frequency facilitation (*n* = 10, Figure 1(b)). In some neurones, facilitation was replaced by a weak frequency-dependent depression of eEPSCs in the presence of the blockers. This can be seen as a reduction in mean amplitude of eEPSCs in the presence of the blockers (e.g., Figure 1(b)). In a further 5 neurones, we confirmed the specificity of the effect by testing the effects of GluR5 subunit specific antagonist of kainate receptors (UBP 302, 20 *μ*M), since we have recently shown that these receptors mediate a similar short-term facilitation of glutamate transmission at 3–5 Hz in layer III of the EC (Chamberlain S.E.L and Jones R.S.G. unpublished). UBP 302 had no effect on facilitation in layer V (not shown) confirming its dependence on NMDAar. Interestingly, 2-AP5 had no effect on frequency facilitation in layer III of the EC (not shown), so although similar short-term plasticity is seen in both layers, its underlying mechanism is lamina-specific.

**Figure 2:**
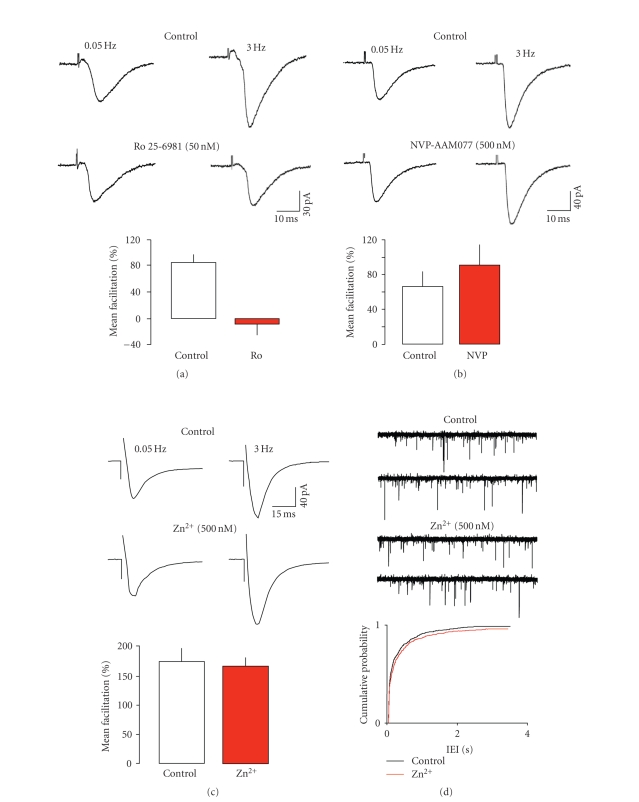
Effects of subunit selective antagonists. (a) Ro 25-6981 abolished frequency-dependent facilitation. In contrast, neither NVP-AAM077 (b) nor Zn^2+^ (c) had any significant effect. (d) Zn^2+^ also had little effect on sEPSCs. The records show consecutive sweeps of baseline recording of sEPSCs and in the presence of Zn^2+^. The cumulative probability plots show pooled data from 6 neurones, with 200 events from each neurone in the presence and absence of the blocker. There was a small shift to the right in the presence of Zn^2+^, but this failed to reach significance (KS test).

Since neither 2-AP5 nor MK-801 has selectivity for NR2A v NR2B subunits [5], the data do not indicate the subunit composition of NMDAr responsible for short-term frequency-facilitation. To determine the receptor involved, we have examined the effect of more specific antagonists. First, we tested the effects of Ro 25-6981. This is an allosteric inhibitor of NMDA receptors, which binds to a site on the N-terminal domain of the NR2 subunit, with a high degree of selectivity (>3000 fold) for NR2B over NR2A [38]. Figure 2(a) shows that Ro 25-6981 at 500 nM abolished the frequency facilitation of eEPSCs, again revealing a weak depression. A lower concentration (200 nM, *n* = 3) of Ro 25-6981 resulted in a mean maximal reduction in frequency-facilitation of 69 ± 7%. At these concentrations, the drug should have little or no effect on NR2A subunits [38], strongly suggesting that NR2B-containing receptors are primarily responsible for this form of short-term plasticity at layer V synapses. This would agree with previous studies that have shown the tonic facilitatory effect on spontaneous release is likely to be NR2B-mediated [16, 35, 43]. Accordingly, Ro 25-6981 resulted in a substantial increase in IEI of sEPSCs from 277 ± 82 milliseconds (5.5 ± 1.9 Hz) to 764 ± 261 milliseconds (2.1 ± 0.7 Hz) recorded in the same neurones (cf. [36, 43]). KS analysis of cumulative probability distributions confirmed a highly significant change. There was no concurrent change in mean amplitude, rise, or decay time (not shown).

Next, we examined the effect of NVP-AAM077 in 5 neurones. This is a competitive antagonist that shows some selectivity for receptors containing the NR2A subtype. Initial reports indicated a greater than 100 fold selectivity of the compound for NR2A over NR2B [39, 44]. However, recently, it has been suggested that the selectivity is closer to 10 fold when the affinity of the two subtypes for glutamate is accounted for ([41], see also [45, 46]). Thus, at the concentration employed here (400 nM), we might expect almost complete blockade of NR2A receptors, but it is possible that substantial inhibition of NR2B would also occur [41]. Nevertheless, NVP-AAM077 did not significantly affect the frequency-dependent facilitation of eEPSCs (see Figure 2(b)). If anything, the facilitation was slightly (although not significantly) increased. These data suggest that NVP-AAM077 may have reasonable selectivity for the NR2A receptor in our preparation, but that these receptors are not involved in presynaptic short-term plasticity at layer V synapses. Further support for this was obtained from analysis of sEPSCs. The mean IEI in control was 443 ± 230 milliseconds (4.0 ± 0.9 Hz), and this decreased slightly to 377 ± 180 milliseconds (4.5 Hz) with the addition of NVP-AAM077. Likewise, there was no change in amplitude, rise, or decay times of sEPSCs (not shown).

In view of the controversy over the selectivity of NVP-AAM077, we also tested (*n* = 5) the effects of Zn^2+^, which has been shown to discriminate between NR2A and NR2B receptors. Like Ro 25-6981 at NR2B subunits, Zn^2+^ binds to the N-terminal domain of the NR2A subunit to exert a voltage-independent inhibition with >100 fold selectivity over NR2B [47–49]. However, as with NVP-AAM077, a relatively high concentration of Zn^2+^ (300 nM) failed to alter frequency-dependent facilitation of eEPSCs (see Figure 2(c)). In addition, it had little effect on the IEI (200 ± 150 *v* 298 ± 170 milliseconds, see Figure 2(d)), amplitude (17.7 ± 3.4 *v* 15.4 ± 2.2 pA), rise (1.9 ± 0.3 *v* 2.1 ± 0.4 milliseconds), or decay times (24.6 ± 1.6 *v* 27.3 ± 1.3 milliseconds) of sEPSCs (cf. [43]). Thus, the data from both NVP-AAM077 and Zn^2+^ studies militate strongly against a role for NR2A receptors in presynaptic frequency-dependent facilitation in layer V of the EC. The ability of Ro 25-6981 to block facilitation strongly indicates that presynaptic plasticity at these synapses is dependent only on NR2B-containing receptors.

**Figure 3:**
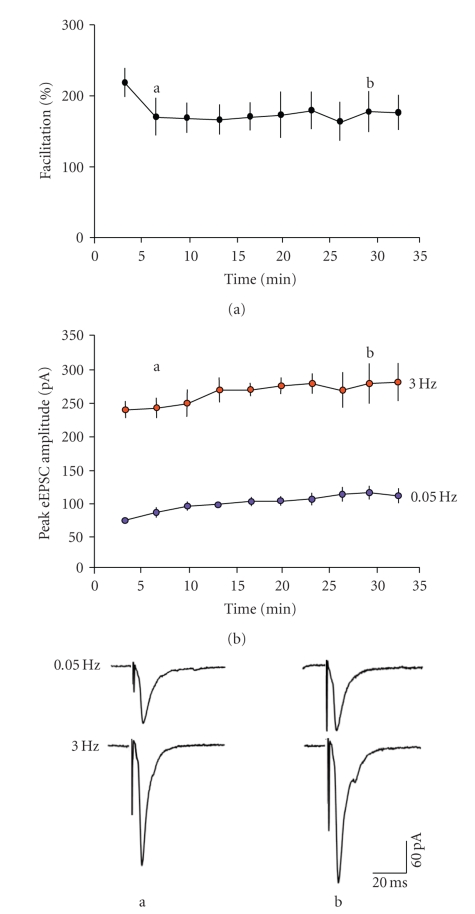
Progressive changes associated with repeated episodes of stimulation at 3 Hz in the absence of NMDAr blockers. Each point is the degree of facilitation recorded during a 30-second period of stimulation and is the average from 5 neurones. (a) After an initial decline in the degree of facilitation, it remained stable throughout the subsequent 30 minutes of recording. (b) Mean amplitude of responses recorded at low and high frequency used to assess the facilitation in the neurones shown in (a). There was a progressive, albeit small increase in amplitude of responses in both cases. Representative records from one neurone, sampled at the times indicated, are shown below.

**Figure 4:**
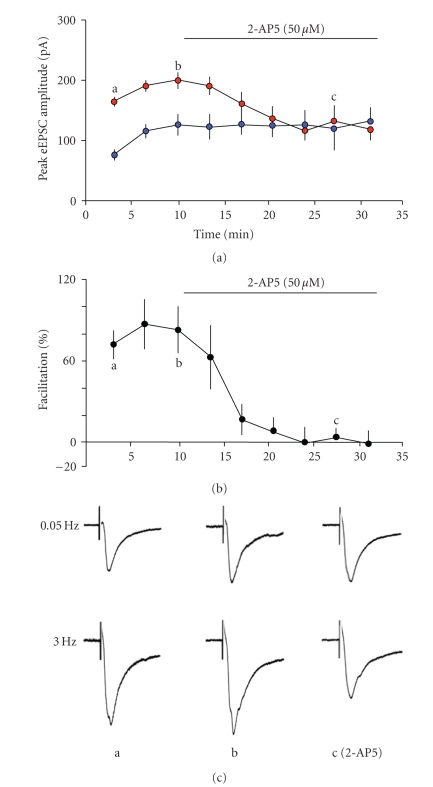
Time course of the effect of 2-AP5 on eEPSC amplitude and facilitation. (a) The progressive increase in both low- and high-frequency responses was prevented by the addition of 2-AP5 (*n* = 5 neurones). The responses at high frequency were progressively reduced to control levels, in parallel with the degree of facilitation (b). (c) Representative responses recorded in one neurone at the times indicated.

A recent paper [50] suggested that activation of postsynaptic NR2B-containing receptors at a similar frequency (3.3 Hz) to that employed by us to elicit frequency-dependent facilitation induced a long-term depression of the NMDAr-mediated currents themselves (primarily by decreasing fractional Ca^2+^ currents carried by the receptors). We were interested to see if the repetitive activation of the presynaptic NR2B-containing receptors would induce any decrement in frequency facilitation at layer V synapses. In 5 neurones, we induced facilitation of eEPSCs and monitored the degree of facilitation but without the addition of any blockers. Overall there was an initial decrease in the degree of facilitation of AMPAr-mediated eEPSCs from the first to second episode, but thereafter it was remarkably consistent (see Figure 3(a)). However, when we looked at absolute amplitude of eEPSCs, there was a small, but consistent, increase over the course of the studies. This applied to events evoked at both low and high frequencies (see Figure 3(b)). We also examined the time course of these changes in the neurones tested with 2-AP5 (see Figure 4). The antagonist appeared to prevent the progressive increase in amplitude of the low-frequency events at the same time as blocking the frequency-dependent facilitation. This limited protocol may suggest the short-term frequency-dependent facilitation could underlie a longer-term enhancement of glutamate transmission. As the postsynaptic NMDAr were already blocked (by internal MK-801), this is likely to involve the presynaptic, NR2B-containing receptors.

### 3.2. Postsynaptic NMDAr

**Figure 5:**
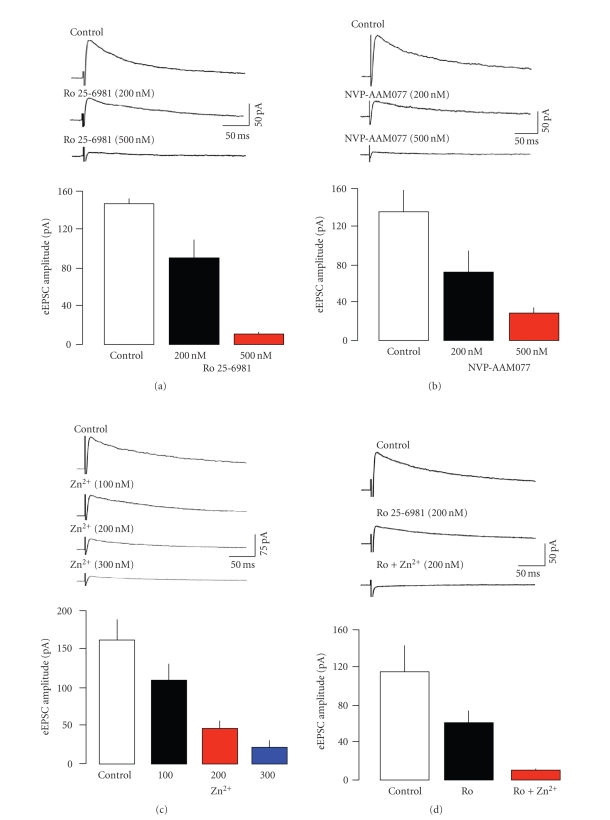
Effect of subunit selective antagonists on postsynaptic NMDAr-mediated eEPSCs. Slow eEPSCs were recorded at +40 mV in the presence of NBQX and bicuculline. Each response is the average of at least 8 events. (a) The NR2B antagonist, Ro 25-691, induced a concentration-dependent reduction in slow eEPSCs. They were essentially abolished at the higher concentration. (b) and (c) show that NR2A selective blockers induced a very similar blockade of slow EPSCs. (d) A combination of NR2A and NR2B antagonists also abolished slow EPSCs.

We now wished to determine the contribution of NR2A/B subunits to NMDAr at postsynaptic sites in layer V of the EC, so we tested the same antagonists used in the presynaptic experiments for effects on isolated NMDAr-mediated eEPSCs. As expected, the nonspecific blockers 2-AP5 (*n* = 5) and MK-801 (*n* = 9) both abolished the slow eEPSCs recorded at +40 mV in the presence of NBQX and bicuculline (not shown). Ro 25-6981 (*n* = 5) also elicited a concentration dependent reduction in postsynaptic NMDAr responses at concentrations that would be expected to retain selectivity for NR2B-containing receptors (see Figure 5(a)). The slow eEPSCs were essentially abolished by Ro 25-6981 at 500 nM. This suggests that NR1/NR2B receptors dominate at postsynaptic sites as they do presynaptically. However, when we tested NVP-AAM077 (*n* = 6), we again found a concentration-related reduction in postsynaptic responses with around 80% inhibition at 500 nM (see Figure 5(b)). Comparison with the data of Neyton and Paoletti [41] suggests that the effect of NVP-AAM077 could be explained by blockade of both NR2B and NR2A receptors since 500 nM was sufficient to abolish NR2A responses in oocytes, but also to exert around 60% block of NR2B. However, this is at odds with its failure to alter preNMDAr-dependent facilitation, which is clearly an NR2B-mediated response. Studies with Zn^2+^ (*n* = 6) failed to substantially clarify the situation. The divalent cation also elicited a concentration-dependent reduction in slow eEPSCs (see Figure 5(c)). The concentrations employed exert around an 80% voltage-independent block of NR2A receptors expressed in oocytes, but retain a considerable degree of selectivity with regard to block of NR2B receptors [47, 49]. These data do suggest a role for NR2A receptors at postsynaptic sites, but it is puzzling that Ro 25-6981 essentially also abolished NMDAr EPSC, when it would be expected to have little effect on NR2A receptors.

We performed two more sets of experiments to look at this question further. In 5 neurones, we first perfused a low concentration of Ro 25-6981 (200 nM), to partially block the NMDAr EPSC. We then added a low concentration of Zn^2+^ (100 nM). In these neurones, Ro 25-6981 resulted in inhibition of around 45%, and with the addition of Zn^2+^ there was a further reduction to around 90–100%, which clearly indicates a role of both NR2A and NR2B in mediating the postsynaptic response (see Figure 5(d)). Finally, there is evidence that under control conditions, NR2A-containing receptors may be substantially blocked by Zn^2+^, present in the ACSF as a result of contamination of other salts used in its preparation [47]. Although addition of Zn^2+^ clearly reduced slow eEPSCs in our experiments, we also examined whether there was significant blockade of the NR2A receptor in control recordings by testing the effect of the Zn^2+^-chelator, TPEN (2 *μ*M), in 3 neurones. This had no effect on the mean amplitude of NMDAr eEPSCs (125.3 ± 25.1 *v* 111.9 ± 26.1 pA) suggesting that our results with antagonists were unlikely to be confounded by Zn^2+^-contamination.

**Figure 6:**
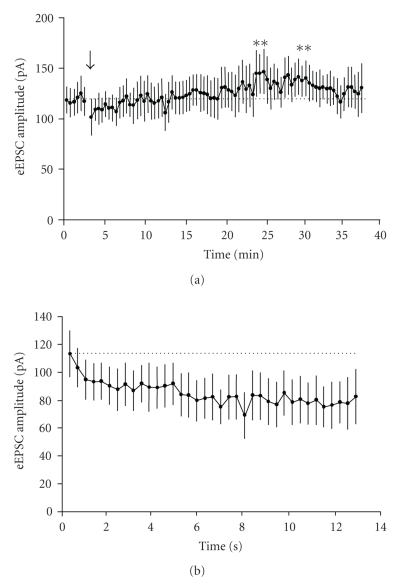
Changes in slow eEPSC amplitudes during and after repetitive stimulation at 3 Hz for 30 seconds. (a) shows the average response amplitudes at low frequency (0.05 Hz) recorded during 35 minutes stimulation in 7 neurones. During the period indicated by the arrow, stimulation was increased to 3 Hz for 30 seconds and the average response amplitudes (first 37 only for clarity) recorded during this period are shown in (b). The only significant differences compared to the mean control value are indicated by the asterisks in (a).

Finally, as noted above, relatively low frequency, repetitive activation of NR2B receptors has been shown to induce a depression of postsynaptic NMDA responses per se [50]. In 7 neurones, we determined the effects of a brief period of repetitive stimulation (3 Hz, 40 seconds) on postsynaptic NMDAr eEPSCs in 5 neurones. Overall, during the repetitive stimulation there was a small (15%), progressive decrease in the first 10–15 seconds, and thereafter the amplitude reached a plateau (see Figure 6(a)). We then recorded NMDAr eEPSCs at low frequency (0.05 Hz) over the subsequent 30 minutes. There was an initial period (5 minutes) where responses appeared to be slightly depressed and thereafter a recovery followed by a slight increase before recovery to control levels (see Figure 6(b)). However, apart from a brief period around 20 minutes there was no significant difference compared to control.

## 4. DISCUSSION

We originally demonstrated that the presynaptic NMDAar mediating facilitation of glutamate release in the EC was likely to be predominantly NR2B-containing, as the frequency of sEPSCs was decreased by the N2B antagonist, ifenprodil [35]. Other work supports the conclusion that preNMDAr that facilitate spontaneous glutamate release at cortical synapses are primarily NR2B-containing. We found that Ro 25-6981 but not NVP-AAM077 or Zn^2+^ reduced sEPSC frequency ([36], present study), and similar results with Ro 25-6981 and Zn^2+^ were reported for synapses in layer II/III of the visual cortex [28]. Jourdain et al. [27] reported that presynaptic NR2B receptors were responsible for the increase in mEPSC frequency in dentate granule neurones seen after stimulation of glutamate release from adjacent astrocytes, as it was blocked by ifenprodil. We now show that the same receptor is likely to mediate short-term plasticity of evoked glutamate release in layer V of the EC. Thus, the facilitation of eEPSCs at the relatively low frequency of 3 Hz was blocked by Ro 25-6981. The lack of effect of NVP-AAM077 and Zn^2+^ suggests that NR2A receptors do not contribute to facilitation of either spontaneous or evoked glutamate release at EC synapses. We cannot rule out a role of NR2A receptors at higher frequencies, although Sjöström et al. [33] have reported that frequency facilitation at 30 Hz at layer V synapses in visual cortex is greatly reduced by ifenprodil, suggesting that NR2B dominate at other presynaptic sites as well.

It is somewhat surprising that only presynaptic NR2B receptors appear to modulate release. Postembedding immunolabeling studies have shown the presence of NR1 subunits in presynaptic terminals in cortex and hippocampus [12–14, 51–53]. Whilst a host of studies have demonstrated NR2B subunits at presynaptic locations [15, 51, 54–59], similar studies have also indicated the presence of NR2A subunits [51, 52, 60–62] although, to date, there are no similar studies specifically related to the EC.

The presence of all three subunits suggests that both NR1/NR2A and NR1/NR2B diheteromeric receptors and possibly also NR1/NR2A/NR2B triheteromers could be expressed in cortical presynaptic terminals, and this may well be the case. However, it is clear from the pharmacological experiments presented here and elsewhere, that NR1/NR2B receptors are predominantly responsible for short-term NMDAr-mediated facilitation of glutamate release (but see, [63]). The properties of NR2B subunits differ from NR2A, in a way that may make them more suited to the task of presynaptic facilitation (see [6, 7, 64–66]). NR2B subunits have a higher affinity for both glutamate and glycine, and show less desensitization. The two subunits confer similar single channel conductance to diheteromeric receptors (around 50 pS), but they have very different deactivation kinetics, with NR1/NR2A receptors having decay time constants of 50–100 milliseconds, and NR1/NR2B receptors in the order of 200–400 milliseconds. Both are Ca^2+^-permeable, but NR2B receptors exhibit a higher fractional Ca^2+^-current than NR2A (see [66, 67]). Both subunits also display Ca^2+^-dependent inactivation, but this is more pronounced for NR2A. The presence of NR2B subunits results in prolonged EPSPs compared to those seen when NR2A subunits dominate (see [3, 7, 66]). Thus, it seems likely that activation of presynaptic NR2B-containing receptors would mediate a slowly deactivating opening of the NMDAr channel and a greater Ca^2+^-influx into the presynaptic terminals than any influx mediated by NR2A receptors. Ca^2+^-influx via the NMDAr is responsible for instantaneous control of spontaneous glutamate release [35]. With a deactivation time of around 300 milliseconds, repetitive activation of NR1/NR2B receptors would readily result in temporal summation of presynaptic Ca^2+^-entry leading to the short-term facilitation at even relatively low-frequency stimulations seen here and previously [35].

It is interesting to speculate on a physiological or pathological role for short-term plasticity mediated by preNMDAr. State-dependent rhythms and oscillatory activity at various frequencies occur in the networks of the EC including ripples and sharp waves (>100 Hz), gamma (30–80 Hz), theta (4–8 Hz), and slow waves (0.1–0.5 Hz) [68–71], and these may be involved in mnemonic processing in temporal lobe structures. There is a consensus that theta oscillations are intimately involved in declarative memory and spatial navigation (see [72–74]), and it is possible that information encoding involved in these processes is reliant on an increase in entorhinal-hippocampal delta/theta coherence [73]. The facilitation of glutamate transmission mediated by preNMDAr that we describe is readily elicited at frequencies in the low theta range. Thus, we could speculate that these receptors may be involved in the generation of theta activity in the EC, and the proposed role of this activity in short-term memory and coding of spatial information (e.g., [72, 74]).

At a pathological level, it is noteworthy that, oscillations at delta (1-2 Hz) and theta frequency may be associated with epilepsy. In patients with temporal lobe epilepsy, there is a generalized increase in EEG activity in the delta/theta range, and the most common pattern of discharges after the initiation of ictal events is a rhythmic delta/theta activity (e.g., [75, 76]). Also, in rats made chronically epileptic following kainic acid injection, epileptiform events in superficial layers of the EC were sometimes followed by spontaneous theta oscillations in layer V [77]. We recently showed that preNMDAr function declines in adulthood, but is markedly enhanced in age-matched, chronically epileptic rats [36] and there is evidence for a similar increased function in human temporal lobe epilepsy [78]. We could speculate that this increased preNMDAr function could result in enhanced generation of delta/theta activity in epileptic conditions. Of further interest in this regard is the observation that increased delta/theta EEG activity (albeit in patients with generalized absence/myoclonic seizures) is normalized by the anticonvulsant drugs, valproate, and lamotrigine [79–81]. We have also shown that at least one anticonvulsant drug (felbamate) can block the preNMDAr [42]. This raises the possibility that some anticonvulsants could alter delta/theta oscillations by targeting preNMDAr.

Whatever the function of short-term plasticity, and the involvement of preNMDAr in it, there is increasing evidence that these receptors may also contribute to longer term forms of plasticity, apparently mediating both LTD [17, 22, 33, 34] and LTP [26, 32] at a variety of synapses. In at least one case, LTD appears to be mediated by NR2B-containing receptors [33], so both short- and long-term plasticity of glutamate transmission could involve Ca^2+^-influx via presynaptic NR2B receptors. We have also shown recently that preNMDAr are rapidly mobile and can diffuse between locations near release sites and more distal locations in the terminal membrane [82]. Trafficking of receptors in the presynaptic membrane appears to be influenced by ongoing activity levels, and exerts an intermediate (over 10 seconds of minutes) form of plasticity. Thus, presynaptic NR2B receptors may be heavily involved in both plasticity and metaplasticity at glutamate synapses in EC and other cortical synapses.

In the present study, we also present evidence for differences in pre- and postsynaptic NMDAr at layer V synapses. Whilst preNMDAr-mediated effects are exclusively dependent on NR1/NR2B-containing diheteromers, both NR2B and NR2A appear to contribute to postsynaptic responses. However, the relative contributions of the two subunits are not clear. The ability of low concentrations of both Zn^2+^ and Ro 25-6981 to reduce postsynaptic NMDAr responses could suggest that they are dependent on a mix of NR1/NR2A and NR1/NR2B diheteromeric receptors. However, concentrations of either blocker, that should largely retain selectivity at the respective subtypes, were able to almost abolish postsynaptic responses. This could suggest that the postsynaptic receptors could be largely triheteromeric NR1/NR2A/NR2B receptors. Although triheteromeric receptors do exhibit high affinity for both NR2A and NR2B selective blockers, it seems likely that they exhibit a reduced maximal inhibitory effect to either, and that maximal blockade requires occupation of both sites [83]. This does not fit well with our finding that combined application of low concentrations of Zn^2+^ and Ro 25-6981 could also abolish postsynaptic responses, which would better support a mediation by a mix of NR1/NR2A and NR1/NR2B diheteromeric receptors. It should also be noted that the ability of NMDA antagonists to block the receptors is not just dependent on the NR2 subunit present, but is also modified by which splice variant of the NR1 subunit with which it combines [47, 49]. We do not know which NR1 subunit(s) may be present in the EC. Thus, overall it is difficult to define exactly what the postsynaptic receptor population, but the most likely scenario is a mix of NR1/NR2A, NR1/NR2B, and NR1/NR2A/NR2B receptors.

A number of studies have suggested that NR1/NR2A, NR1/NR2B, and NR1/NR2A/NR2B receptors may contribute to postsynaptic responses at other cortical synapses [84–86]. There is support also for synapse-specific segregation of NR2A and NR2B-containing receptors (e.g., [87, 88]) and spatial segregation between subsynaptic and extrasynaptic sites (e.g., [86]). The controversy over whether subunit composition and spatial location are linked, and the difficulties in defining the role of triheteromeric receptors has been well reviewed recently [3]. We cannot make any firm conclusions regarding these aspects in the EC, but our data do suggest that postsynaptic NR1/NR2A, NR1/NR2B, and NR1/NR2A/NR2B receptors all contribute to postsynaptic responses at glutamate synapses in layer V of the EC, in contrast to presynaptic sites where NR1/NR2B receptors may have exclusive control. Increasing numbers of studies have documented LTP and LTD at synapses in the EC [89–95]. The EC is clearly a pivotal site in learning and memory functions resident in the temporal lobe. We have shown that preNMDAr mediate short-term forms of plasticity in the EC. In experiments employing a limited protocol of repetitive activation, we found that this short-term plasticity may lead to longer-term plasticity (either pre- or postsynaptically), and the aim now is to examine in detail the relationship between short-term effects and long-term plasticity and metaplasticity at these synapses.
